# Unearthing of Key Genes Driving the Pathogenesis of Alzheimer’s Disease via Bioinformatics

**DOI:** 10.3389/fgene.2021.641100

**Published:** 2021-04-16

**Authors:** Xingxing Zhao, Hongmei Yao, Xinyi Li

**Affiliations:** ^1^Department of Neurology, Bethune Hospital Affiliated to Shanxi Medical University, Taiyuan, China; ^2^Department of Cardiology, First Hospital of Shanxi Medical University, Taiyuan, China

**Keywords:** WGCNA, Alzheimer’s disease, pathogenesis, machine learning, biomarkers

## Abstract

Alzheimer’s disease (AD) is a neurodegenerative disease with unelucidated molecular pathogenesis. Herein, we aimed to identify potential hub genes governing the pathogenesis of AD. The AD datasets of GSE118553 and GSE131617 were collected from the NCBI GEO database. The weighted gene coexpression network analysis (WGCNA), differential gene expression analysis, and functional enrichment analysis were performed to reveal the hub genes and verify their role in AD. Hub genes were validated by machine learning algorithms. We identified modules and their corresponding hub genes from the temporal cortex (TC), frontal cortex (FC), entorhinal cortex (EC), and cerebellum (CE). We obtained 33, 42, 42, and 41 hub genes in modules associated with AD in TC, FC, EC, and CE tissues, respectively. Significant differences were recorded in the expression levels of hub genes between AD and the control group in the TC and EC tissues (*P* < 0.05). The differences in the expressions of *FCGRT*, *SLC1A3*, *PTN*, *PTPRZ1*, and *PON2* in the FC and CE tissues among the AD and control groups were significant (*P* < 0.05). The expression levels of *PLXNB1*, *GRAMD3*, and *GJA1* were statistically significant between the Braak NFT stages of AD. Overall, our study uncovered genes that may be involved in AD pathogenesis and revealed their potential for the development of AD biomarkers and appropriate AD therapeutics targets.

## Background

Alzheimer’s disease (AD) is a type of dementia, which is commonly associated with β-amyloid and neurofibrillary tangles (NFTs). Many clinical trials have faced difficulties in reducing β-amyloid (Ashraf and So, 2020). The main characteristics of AD are the formation of NFTs, synapse loss, and the deposition of senile plaques (Gouras et al., 2000; Patnaik et al., 2020). Several studies reported that the distribution of NFTs in the brain is highly associated with cognitive impairment status in AD (Braak and Braak, 1991; Nelson et al., 2012). Braak NFT stages refer to the six stages (I, II, III, IV, V, and VI) of the development of NFTs according to the spatial distribution of tangle-bearing neurons in the brain (Braak et al., 2006a). Whether a large amount of tau protein can be detected in different parts of the AD brain is determined by Braak NFT stages. Generally, the diagnosis sites include entorhinal regions (stages I–II), limbic allocortex and adjoining neocortex (stages III–IV), and neocortex (stages V–VI) (Braak et al., 2006b). So far, the pathogenesis of AD remains mostly unclear, although several theories have been proposed to explain AD pathogenesis, including tau pathology, oxidative stress, cholinergic neurodegeneration, neuroinflammation, and amyloidosis (Agostinho et al., 2010; Hampel et al., 2018). Nevertheless, treatment methods derived from existing theories still cannot effectively limit the growing number of AD patients. Therefore, to discover new pharmacological targets, it is urgent to identify the molecular basis of the disease.

The weighted gene coexpression network analysis (WGCNA) is a widely used method to discover complex relationships between modules and traits (Langfelder and Horvath, 2008). The primary function of WGCNA is that it can gather genes with similar expression levels into a module according to the correlation coefficient between genes; thus, it can be used to analyze the relationship between modules and sample traits. WGCNA links sample traits and gene expression alteration, facilitating an in-depth understanding of the systematic signaling networks correlated with phenotypes of interest (Ma et al., 2017). Previous studies have adopted WGCNA to find significant genes and study the pathological mechanism. Changes in peripheral blood may participate in AD pathology, and research of AD using whole blood (WB) samples suggested that *ATF4*, *TRPV2*, *HSPA8*, *NDUFV1*, *LUC7L3*, and *STAT3* may contribute to the occurrence of MCI (Tang and Liu, 2019). Except for whole-blood samples, mouse (Rangaraju et al., 2018; Mukherjee et al., 2019; Pandey et al., 2019), *Caenorhabditis elegans* (Godini et al., 2019), and zebrafish (Hin et al., 2020) with AD have been studied widely by scientists. Moreover, WGCNA and PCA were used to reveal the unique transcriptome signatures among chronic traumatic encephalopathy (CTE), CTE/AD, and AD (Cho et al., 2020).

This study aimed to explore and find key pathways involved in AD as well as to identify potential genes related to AD pathogenesis. We constructed WGCNA using 267 samples, including 100 control and 167 AD samples from four tissues [temporal cortex (TC), frontal cortex (FC), entorhinal cortex (EC), and cerebellum (CE)]. Then we identified key modules associated with AD and further analyzed modules with high correlation. Next, we uncovered hub genes by Cytoscape MCODE plugin. Additionally, we compared the expression levels of differentially expressed genes (DEGs) and hub genes and figured out significant genes involved in AD. Finally, we obtained the expression of hub genes in GSE131617 datasets and verified the classification function of hub genes based on AD classifiers. For the first time, we compared the expression levels of hub genes in four tissues of the brain and obtained the potential pathway associated with AD development in these tissues. Our results found that the expression levels of *PLXNB1* and *GJA1* based on Braak NFT stages were significant, suggesting that these genes may be involved in AD pathogenesis and have a high potential for the development of AD biomarkers and target drugs.

## Materials and Methods

### Data Collection and Data Preprocessing

The AD datasets of GSE118553 and GSE131617 were collected from the NCBI GEO datasets^1^. GSE118553 contained 167 AD samples, 100 control samples, and 134 asymptomatic AD (AsymAD) samples (Table 1). We divided the GSE118553 dataset into four groups according to the source of the samples (Table 1). GSE131617 consisted of 426 brain tissue specimens, which were generated from three regions (TC, FC, and EC) of the brain. Here, we used the GPL5175 of the GSE131617 dataset, which was divided into four groups according to the Braak NFT stage. The corresponding information can be seen in Table 2. The Python scripts were used to process the raw data and generate the gene matrix, and the scripts are available on GitHub^2^. The average expression level of a gene was retained if the gene mapped with multiple probes. The expression data of the gene matrix was log2 transformed as in the previous studies (Ambroise et al., 2011; Ritchie et al., 2015).

**TABLE 1:** The information of GSE118553.

**Sample source**	**AD**	**Control**	**AsymAD**
Temporal cortex (TC)	52	31	32
Frontal cortex (FC)	40	23	33
Entorhinal cortex (EC)	37	24	37
Cerebellum (CE)	38	22	32
Sum	167	100	134

**TABLE 2:** The information of GSE131617 GPL5175.

**Braak NFT stage**	**Sample source**	**Samples**
0	13 TC; 13 FC; 13 EC	39
I–II	20 TC; 20 FC; 20 EC	60
III–IV	19 TC; 19 FC; 19 EC	57
V–VI	19 TC; 19 FC; 19 EC	57
–	–	213

*TC, temporal cortex; FC, frontal cortex; EC, entorhinal cortex; CE, cerebellum.*

### Construction of Weighted Gene Coexpression Network Analysis

The matrix data of GSE118553 were obtained from the GEO database. We kept the mean value of gene expression when a gene matched with multiple probes and finally chose 31,413 genes for WGCNA. We used a step-by-step construction method for the coexpression network and chose soft power based on the pickSoftThreshold function. This function can provide a suitable β value by calculating the scale-free topology fit index for a series of powers. Then, an adjacency matrix was constructed based on soft-thresholding powers and transformed into a topological overlap. A hierarchical clustering function was applied to cluster genes with similar expression levels into several modules according to the 1-TOM. Each module was represented by the module eigengene (ME), which refers to the PC1 of the expression level in the genes from a module. Dynamic tree cut was used to reduce the number of modules according to the dissimilarity of MEs, and the cutoff value was 0.25. The traits in this study included AD and gender. The correlation between MEs and AD was calculated for key module selection based on Pearson correlation coefficient (PCC). The function corPvalueStudent was used to calculate the Student asymptotic *P*-value. Except for the gray module, we chose the modules with the highest and lowest correlation with AD as key modules (*P* < 0.05). The WGCNA was completed by the R package “WGCNA” (Langfelder and Horvath, 2008, 2012). The biological function of genes in key modules associated with AD was investigated by the functional enrichment analysis described below.

### Uncovering of Hub Genes in the Significant Module and Functional Enrichment Analysis

In the coexpression network, the nodes with high interconnection in a key module were defined as hub genes. First, we calculated module membership (MM) (also refers to the correlation between ME and gene expression) and the gene significance (GS) (also refers to the correlation between gene expression and traits). As the description of the author of WGCNA (Langfelder and Horvath, 2008), the genes with a higher MM in the trait-specific modules were considered as candidate genes for further validation. Meanwhile, a higher mean GS of a module indicates the more significant the correlation between the module and traits. The significant genes were identified according to the threshold of MM ≥ 0.8 and GS ≥ 0.2 as suggested by previous studies (Liang et al., 2020; Mo et al., 2020; Xia et al., 2020). Second, we selected the top 299 edges of the significant genes by weight in each module for Cytoscape visualization and finally got a weighted network of key modules. Additionally, a subnetwork of the co-weighted network was generated by the Cytoscape MCODE plugin with the default configuration. Finally, we selected the nodes clustered in the subnetwork as hub genes and performed functional enrichment analysis as described below.

### Screening of DEGs and Functional Enrichment Analysis

To further understand the gene expression of AD and control groups in GSE118553 in different tissues, the gene expression matrix of GSE118553 was divided into four tissue-specific gene expression matrices according to the tissues. Based on each tissue-specific gene expression matrix, the DEG analysis was performed between the AD and control groups based on the R package “limma” (Ritchie et al., 2015). The normalization was completed by R package “limma.” The Benjamini–Hochberg (BH) method was used to calculate the *adj.P*-value and was implemented by the R package “limma.” The DEGs were screened out based on *adj.P*-value < 0.05 and abs(log2FoldChange) > 1.2 as in previous studies (Yang et al., 2014; Richard et al., 2016). The top 50 upregulated and downregulated DEGs were selected based on | log2FoldChange| and visualized as a heatmap by the R package “pheatmap”^3^. Moreover, the function and pathway of DEGs were uncovered by functional enrichment analysis as described below. The overlap of DEGs, modules genes, and hub genes were visualized by the R package “UpSetR” (Conway et al., 2017).

### Functional Enrichment Analysis

The function and pathway of genes from key modules, hub genes, and DEGs were analyzed by Gene Ontology (GO) analysis by using R packages “clusterProfiler” (Yu et al., 2012). The top 15 GO terms including biological process (BP), cellular component (CC), and molecular function (MF) of the key modules were shown in the bubble chart. Meanwhile, the top 10 KEGG pathways of the key modules were also visualized by the bubble chart. The BH method was carried out for multiple testing.

### Validation of Significant Hub Genes by Machine Learning

The significant hub genes were defined as the intersection of hub genes and DEGs in this study. To validate the AD classification function of significant hub genes, we constructed AD classifiers using several machine learning algorithms from the scikit-learn library (Pedregosa et al., 2011). The expression data of significant hub genes in GSE118553 were used for training and testing of the AD classifier. A total of 10 standard algorithms, including support vector machine (SVM), random forest (RF), extra tree, adaptive boosting (AdaBoost), gradient boosting, multi-layer perceptron (MLP), K-nearest neighbors (KNN), logistic regression, linear discriminant analysis, and Gaussian Naive Bayes classifier (Gaussian NB), were used in this study. First, we divided the GSE131617 into two parts according to 70% training and 30% testing and selected 10 machine learning algorithms for AD classifiers according to the suggestion of previous studies (Kringel et al., 2018; Tunvirachaisakul et al., 2018; Xu et al., 2018; Chen et al., 2019; Shigemizu et al., 2019; So et al., 2019; Bi et al., 2020; Yaman et al., 2020). Then we optimized the AD classifiers through parameter adjustment and selected 30% of the data as the test. Finally, we generated the receiver operating characteristic (ROC) curve of the AD classifiers and calculated the area under the curve (AUC) to distinguish the performance of AD classifiers. The detailed information of the machine learning classifiers is summarized in Table 3. The classification metrics including F1 score, sensitivity, specificity, PPV, and NPV were calculated as in previous studies (Chen et al., 2009; Ray et al., 2010; Trevethan, 2017).

**TABLE 3:** The hyperparameter tuning of 10 machine learning classifiers.

**Classifiers**	**Parameter**
SVM	kernel = ‘linear’, probability = True, Random_state = 42
Random forest	Random_state = 42
Extra trees	Random_state = 42
AdaBoost	DecisionTreeClassifier (random_state = 42), Random_state = 42, learning_rate = 0.1
Gradient boosting	Random_state = 42
MLP classifier	Random_state = 42
K neighbors	Default configuration
Logistic regression	Random_state = 42
Linear discriminant analysis	Default configuration
Gaussian NB	Default configuration

*The number of cross-validation of all machine learning classifiers was 6.*

Moreover, we validated the expression levels of hub genes in GSE131617. As mentioned above, Braak NFT stages of AD included different areas of the brain (TC, FC, and EC). Therefore, we determined the role of hub genes in Braak NFT stages through the expression levels of three regions (TC, EC, and FC) in the AD brain. First, the GSE131617 were divided into three groups according to the tissues, including TC, FC, and EC tissues. Then we performed an analysis of variance (ANOVA) of each tissue based on Braak NFT stages of AD. FDR was used to adjust the *P*-value calculated by ANOVA. A Python script was used to perform the ANOVA, which was uploaded on GitHub^4^. The expression of hub genes in different tissues was visualized by beeswarm plot and was implemented by using the R package “beeswarm”^5^.

## Results

### Construction of Weighted Gene Coexpression Network Analysis

**FIGURE 1:**
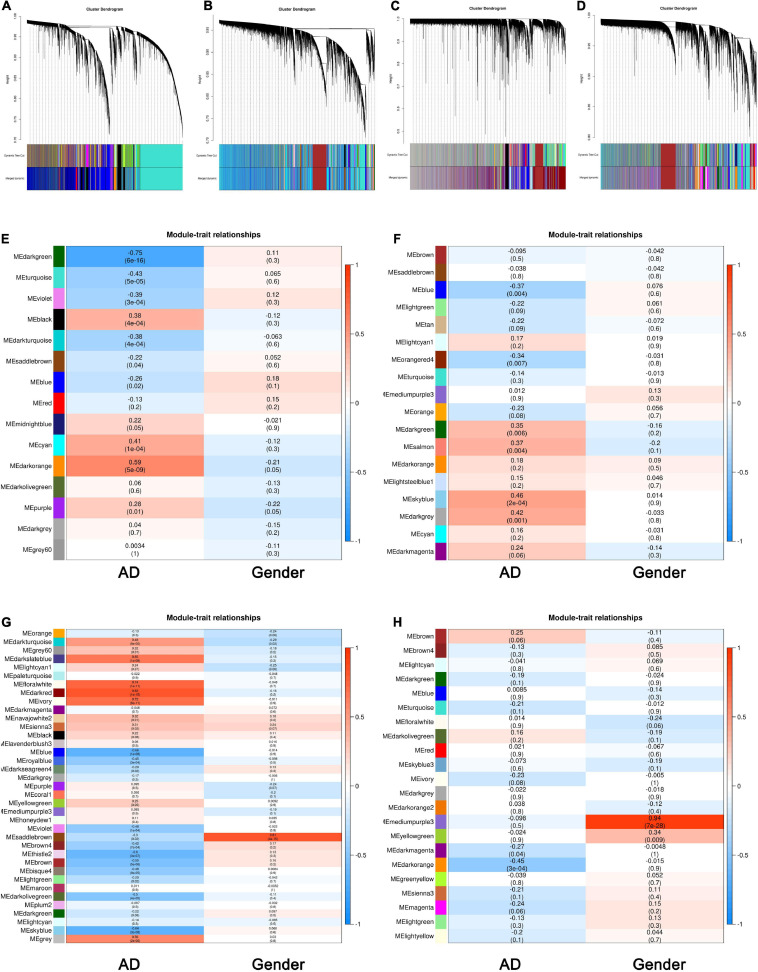
Identification of key modules associated with Alzheimer’s disease (AD) in the temporal cortex (TC), frontal cortex (FC), entorhinal cortex (EC), and cerebellum (CE) tissues. **(A–D)** Clustering dendrogram of genes in the TC **(A)**, FC **(B)**, EC **(C)**, and CE **(D)** tissues, with gene dissimilarity based on 1-TOM, together with module color assignment. Different colors represent the different modules in which genes are gathered. The dynamic tree cut method was used to merge the modules according to the dissimilarity of module eigengenes (MEs). **(E)** Correlation among modules and traits in the TC tissue. **(F)** Correlation among modules and traits in FC tissue. **(G)** Correlation among modules and traits in EC tissue. **(H)** Correlation among modules and traits in CE tissue. The box includes the Pearson correlation coefficient (PCC) and the corresponding *P*-value. The box color (ranges from blue to red) is correlated with the PCC value (range from –1 to 1). The red box indicates the module is positively correlated with traits, while the blue box indicates the module is negatively correlated with traits. The traits in this study include AD and gender.

We chose an appropriate β value for WGCNA via analyzing the topology of the network. As shown in Supplementary Figures 1A–C, when the β value was 3, and the dissimilarity of MEs equaled 0.25, a total number of 31,413 genes were clustered into 15 modules in the TC tissue. The same treatment was applied to the genes in the FC, EC, and CE tissues. The most appropriate power values were 2, 9, and 2, respectively, for the FC, EC, and CE tissues (Supplementary Figures 2–4A–C). The dendrogram plot of all genes grouped depending on 1-TOM and clustering of MEs is shown in Figures 1A–D.

To have a deeper understanding of the correlation between modules and AD, we obtained the correlation between MEs and sample traits, including AD and gender. As shown in Figure 1E, MEdarkorange (cor: 0.59, *P*-value: 5e−09) and MEdarkgreen (cor: −0.75, *P*-value: 6e−16) were the most significant modules among the modules associated with AD in the TC tissue. Figure 1F shows MEblue (cor: −0.37, *P*-value: 0.004) and MEskyblue (cor: 0.46, *P*-value: 2e−04) were the most significant modules among the modules associated with AD in the FC tissue. Figure 1G indicates that MEblue (cor: −0.66, *P*-value: 1e−08) and MEdarkred (cor: 0.82, *P*-value: 1e−15) were the most significant modules among the modules associated with AD in the EC tissue. As shown in Figure 1H, MEdarkorange (cor: −0.45, *P*-value: 3e−04) and MEbrown (cor: 0.25, *P*-value: 0.06) were the most significant modules among the modules associated with AD in the CE tissue. The PC1 (ME) of modules identified by WGCNA is summarized in Supplementary Table 1.

### Function Annotation of the Significant Modules

**FIGURE 2:**
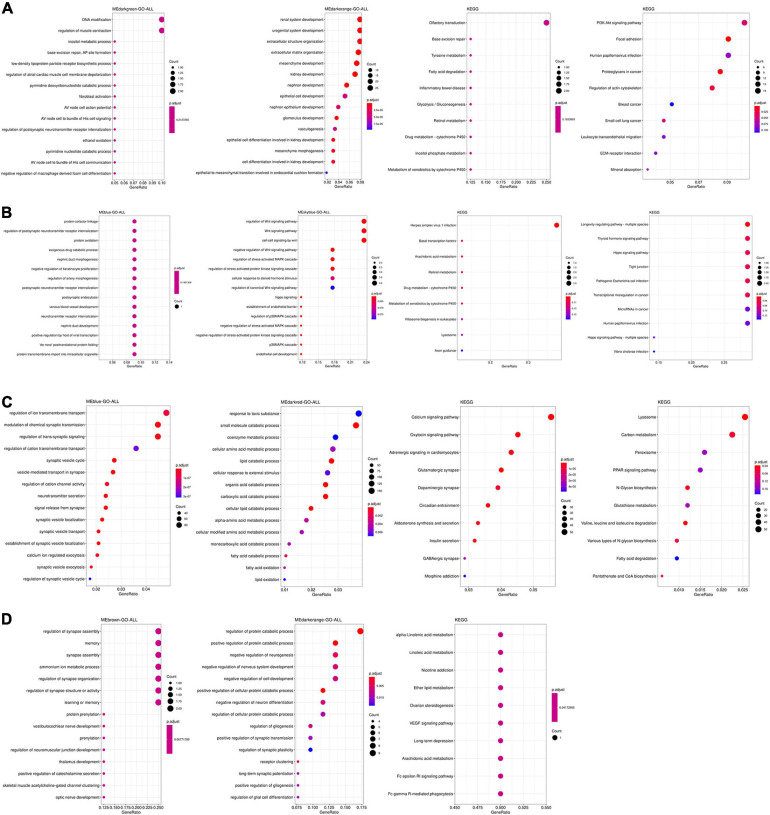
Functional enrichment analysis of genes from significant modules associated with AD in the TC **(A)**, FC **(B)**, EC **(C)**, and CE **(D)** tissues. The color of the bubble indicates the *adj.P*-value of the GO terms or pathways, and the size of the bubble signifies the number of genes associated with a term.

The function and pathways of genes from significant modules associated with AD in the four tissues were analyzed by functional enrichment analysis. As shown in Figure 2A, genes from significant modules associated with AD in the TC tissue were enriched in renal system development, nephron development, DNA modification, focal adhesion, PI3K–Akt signaling pathway, metabolic process, and tyrosine metabolism. Figure 2B showed that genes from significant modules associated with AD in the FC tissue were enriched in the carboxylic ester hydrolase activity, focal adhesion, and mineral absorption. Figure 2C indicated that genes from significant modules associated with AD in the EC tissue were enriched in the modulation of chemical synaptic transmission, synaptic vesicle cycle, and synaptic vesicle transport. The results shown in Figure 2D revealed that genes from significant modules associated with AD in the CE tissue were involved in the regulation of synapse assembly, memory, learning or memory, optic nerve development, and long-term depression.

### Identification and Function Annotation of Hub Genes of Key Modules

According to the criteria MM ± 0.8 and GS ± 0.2, we obtained significant hub genes associated with AD in the four tissues (Supplementary Figures 1–4D–E). Then, hub genes were clustered via Cytoscape MCODE (Supplementary Figures 1–4F–G), and the expression levels of hub genes are shown in Supplementary Figures 5–8. The detailed information of the edges in the co-weighted network of each module is summarized in Supplementary Table 2. We obtained 33, 42, 42, and 40 hub genes, respectively, in the TC, FC, EC, and CE tissues.

**FIGURE 3:**
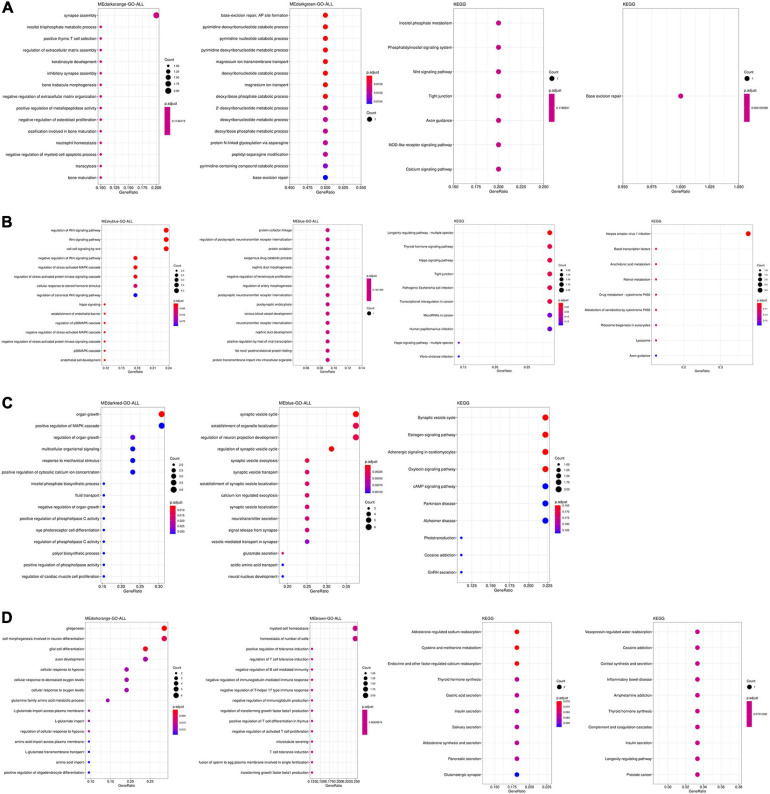
Functional enrichment analysis of hub genes in the TC **(A)**, FC **(B)**, EC **(C)**, and CE **(D)** tissues. The color of the bubble indicates the *adj.P*-value of the GO terms or pathways, and the size of the bubble signifies the number of genes associated with a term.

The function and pathway of hub genes from significant modules associated with AD in the four tissues were analyzed. In the TC tissue (Figure 3A), the hub genes were enriched in synapse assembly, neutrophil homeostasis, DNA modification, cognition, and actin filament organization. Figure 3B showed that the hub genes from significant modules associated with AD in FC tissue were enriched in the protein–cofactor linkage, regulation of postsynaptic neurotransmitter receptor internalization, and protein oxidation. Figure 3C suggests that the hub genes from significant modules associated with AD in EC tissue were enriched in the synaptic vesicle cycle, synaptic vesicle localization, and signal release from synapse. The hub genes from significant modules associated with AD in the CE tissue were enriched in myeloid cell homeostasis, homeostasis of a number of cells, and regulation of T-cell tolerance induction (Figure 3D).

### Identification and Function Annotation of the DEGs

Differentially expressed genes were filtered out by the threshold *adj.P*-value < 0.05 and abs(log2FoldChange) > 1.2. Finally, we obtained 3,648 DEGs in the TC tissue, 555 DEGs in the FC tissue, 6,504 DEGs in the EC tissue, and 188 DEGs in the CE tissue. The DEGs identified in the four tissues were visualized on volcano plots (Supplementary Figures 9A–D), and the top 10 DEGs in upregulated and downregulated clusters (ranked by *adj.P*-value) were labeled. The top 25 DEGs in upregulated and downregulated clusters (sorted by | log2FoldChange| are shown in Supplementary Figures 9E–H. The corresponding details of DEGs identified in four tissues are found in Supplementary Table 3.

**FIGURE 4:**
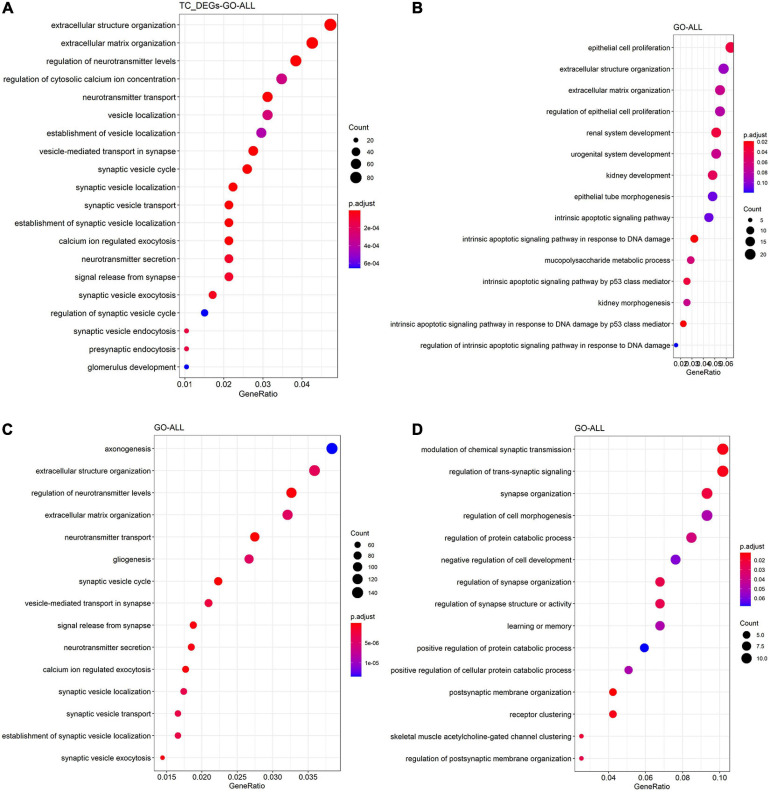
Functional enrichment analysis of DEGs in the TC **(A)**, FC **(B)**, EC **(C)**, and CE **(D)** tissues. The color of the bubble indicates the *adj.P*-value of the GO terms or pathways, and the size of the bubble signifies the number of genes associated with a term.

To get a better understanding of the biological function of DEGs, we also did GO and KEGG functional analysis. As shown in Figure 4A, DEGs in TC tissue were enriched in regulation of neurotransmitter levels, neurotransmitter transport, regulation of actin cytoskeleton, phagosome, and synaptic vesicle cycle. Figure 4B shows that DEGs in the FC tissue are enriched in epithelial cell proliferation, extracellular matrix organization, and glycolysis/gluconeogenesis. DEGs in the EC tissue are enriched in axonogenesis, regulation of neurotransmitter levels, neurotransmitter transport, synaptic vesicle cycle, neurotransmitter secretion, and synaptic vesicle localization (Figure 4C), while DEGs in the CE tissue are enriched in the postsynaptic membrane organization, synapse organization, and modulation of chemical synaptic transmission (Figure 4D).

**FIGURE 5:**
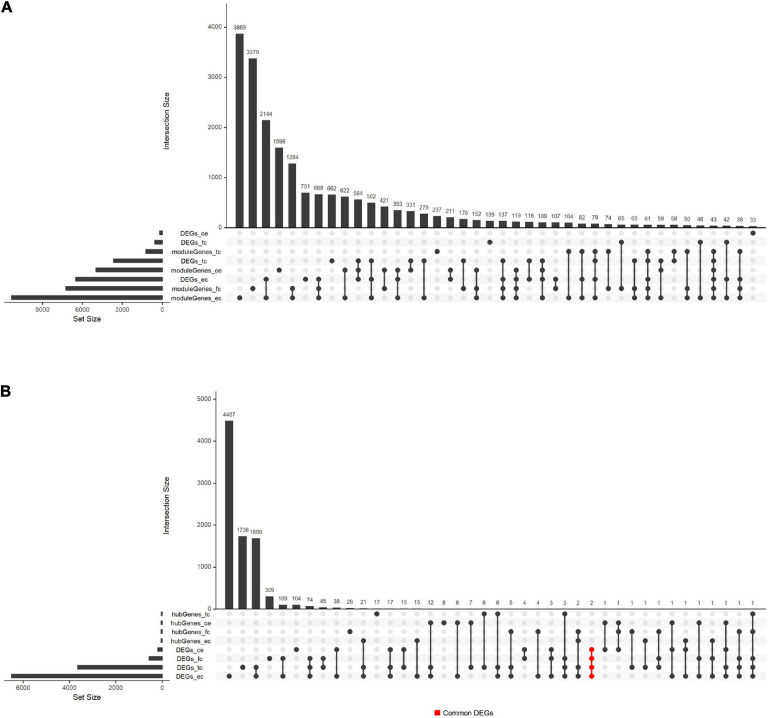
Overlaps between hub genes, DEGs, and module genes in GSE118553. **(A)** The overlap between module genes and DEGs in GSE118553. **(B)** The overlap between hub genes and DEGs in GSE118553. Each column indicates the gene set, and each row indicates the gene names. The light-gray cells indicate that the gene names were not part of that intersection, and the black filled indicates the gene names were part of that intersection. The abbreviations of the row refer to the hub genes, DEGs, and module genes detected in GSE118553. The abbreviations with “moduleGenes” or “hubGenes” refer to the genes obtained in key modules, which represent module genes or hub genes. The abbreviations with “DEGs” refer to the DEGs detected in AD compared with the control group. The abbreviations with “_tc,” “_fc,” “_ec,” or “_ce” indicate that the identified genes are from TC, FC, EC, or CE tissues. The cells in red represent the genes that were the part of the DEGs detected in four tissues and the intersection of them were two genes, which were marked as “Common_DEGs.”

Besides, we compared the overlap of DEGs, hub genes, and module genes. The results shown in Figure 5 suggested that the hub genes from four tissues were relatively independent and did not intersect. The common DEGs were found in four tissues, including *SLC6A12* and *P8*.

### Validation of Hub Genes

**FIGURE 6:**
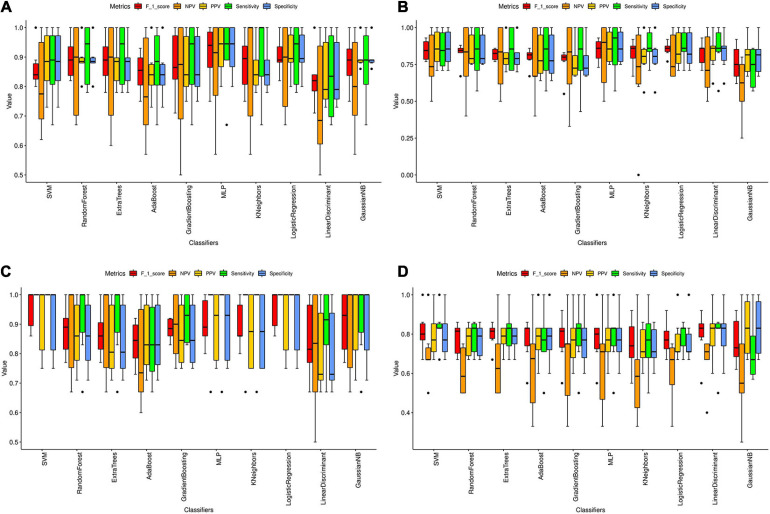
Classification metrics of AD classifiers based on the significant hub genes from modules associated with AD in four tissues. **(A–D)** The classification metrics of 10 AD classifiers based on the significant hub genes detected in TC **(A)**, FC **(B)**, EC **(C)**, and CE **(D)** tissue. The classification metrics include F_1_score, NPV, PPV, sensitivity, and specificity. The average value of each metric in six cross-validations was calculated and represented as the black line in the boxplot. Error bar is provided in the boxplot as well. The black dots refer to the outliers.

We found a total of 33, 3, 42, and 4 significant hub genes in TC, FC, EC, and CE tissues (Supplementary Table 4). To verify the AD classification function of significant hub genes identified above, we constructed AD classifiers using significant hub genes as features. The training and testing data are as indicated in Supplementary Table 4. In TC tissue (Supplementary Figure 10A), the AD MLP classifier had the highest AUC (average of AUC = 0.97 ± 0.05), and the AD AdaBoost classifier had the lowest AUC (average of AUC = 0.78 ± 0.09). In the FC tissue (Supplementary Figure 10B), the AD logistic regression classifier had the highest AUC (average of AUC = 0.86 ± 0.07), while the AD AdaBoost classifier had the lowest AUC (average of AUC = 0.70 ± 0.11). In the EC tissue (Supplementary Figure 10C), the AD SVM classifier had the highest AUC (average of AUC = 0.99 ± 0.00), whereas the AD AdaBoost classifier had the lowest AUC (average of AUC = 0.79 ± 0.09). In the CE tissue (Supplementary Figure 10D), the AD SVM classifier had the highest AUC (average of AUC = 0.85 ± 0.12), while the AD AdaBoost classifier has the lowest AUC (average of AUC = 0.70 ± 0.14). The other classification metrics (F1 score, sensitivity, specificity, PPV, and NPV) of AD classifiers for four tissues are found in Figure 6 and Supplementary Table 5. Consistent with the AUC of AD classifiers in four tissues, we found that the value of the metrics of AD classifiers was higher in TC and EC tissues than in FC and CE tissues.

Additionally, we analyzed the expression levels of hub genes in GSE131617 for validation. Supplementary Figure 11 showed that the expression difference of *PLXNB1* among Braak NFT stages of the AD in TC tissue was significant (*P* < 0.05). No difference was recorded in the expression levels of hub genes based on the Braak NFT stages of the AD in FC tissue (Supplementary Figure 12). The expression of *GJA1* and *GRAMD3* (Supplementary Figure 13) based on Braak NFT stages of AD in EC tissue was significantly different (*P* < 0.05). The detailed results of ANOVA analysis of gene expression among Braak NFT stages are as in Supplementary Table 6.

## Discussion

Alzheimer’s disease is a disease characterized by degenerative changes in the central nervous system (CNS) and is common in the elderly. In this work, we used systems biology analysis methods to mine the potential information of AD transcriptome data sets. A total of 31,413 genes were obtained after data processing and were used for WGCNA analysis. In the TC, FC, EC, and CE tissues, genes were clustered into 15, 18, 37, and 22 gene modules, respectively. Moreover, we identified 33, 42, 42, and 41 hub genes, respectively, in modules significantly associated with AD in TC, FC, EC, and CE tissues. The difference in the expression of hub genes from modules associated with AD in the TC and EC tissue among the AD and control groups were significantly different. Expression of *FCGRT* from modules associated with AD in the FC tissue was significantly different among AD and the control group. Significant differences were recorded in the expression of *SLC1A3*, *PTN*, *PTPRZ1*, and *PON2* from modules associated with AD in CE tissue.

*SLC1A3* is one of the high-affinity glutamate transporters that mediate the cellular uptake of glutamate, resulting in the pathogenesis of AD when the transporters dysfunction (Kanai et al., 2013). A previous work conveyed that *PTN* and *PON2* are involved in AD (Janka et al., 2002; Shi et al., 2004; Xu et al., 2014; Gurung and Bhattacharjee, 2018). However, *PTPRZ1* was rarely reported in past research and deserved a more in-depth study. Exposure of the developing brain to immune mediators promotes neurodevelopmental disorders and neurodegenerative diseases; a previous study reported that IgG antibodies may affect normal neurological development and function through Fc gamma receptors (FcγR) expressed in the hippocampus and cortex of newborn brains (Stamou et al., 2018). *FCGRT* is the fragment of IgG receptor and transporter, which is believed to be related to IgG in the brain (Glass et al., 2017). Therefore, we speculated that *FCGRT* may be involved in AD through the regulation of neural development by IgG antibodies. *PTPRZ1* refers to the receptor-type tyrosine-protein phosphatase zeta and is reported to be expressed in the CNS (Wang et al., 2010). Moreover, PTPRZ1 is a potential schizophrenia susceptibility gene as reported by a previous study (Buxbaum et al., 2008), which may be related to the working memory deficits in mice (Takahashi et al., 2011). Thus, we speculate that *PTPRZ1* may regulate the cognitive and memory pathways through the CNS, thereby promoting the formation of AD.

In recent years, several methods have been proposed in the analysis of the AD dataset. At present, several studies have used bioinformatics methods to mine transcriptome data in different regions of the AD brain. Wang et al. (2020) analyzed the AD dataset (80 AD samples and 28 control samples) of the temporal and dorsolateral prefrontal cortex through three algorithms, and also used computational deconvolution methods to identify differential genes in a single cell type (CI-DEG). Single-cell sequencing has the advantage of uncovering the heterogeneity among individual cells, but often, fewer cases are covered. Wang and Wang (2020) conducted a comprehensive analysis of the AD gene expression dataset, and identified DEGs in hippocampus tissue (HIP), temporal gyrus tissue (TG), frontal gyrus tissue (FG), and WB through differential expression analysis. They found that *GJA1* is a key gene of the PPI network in HIP, TG, and FG, and this study found that *GJA1* is a DEG among AD and control groups in EC tissues. In addition, Wang and Wang (2020) did not use a coexpression network to explore the relationship between gene expression and AD, which may lead to insufficient understanding of the AD dataset. TMEM106B acts as a genetic modifier for the cognitive trajectory in Parkinson’s disease (Tropea et al., 2019). To explore the precise mechanism of neurodegeneration caused by TMEM106B haplotypes, the researchers used ANOVA to obtain DEGs and WGCNA to identify key genes in AD (Ren et al., 2018). However, using only differential genes to build WGCNA may overlook the role of some low-expressed genes. This study used all genes to construct WGCNA, which can effectively avoid such problems. Also, current gene mining on AD mainly uses mice (Rangaraju et al., 2018; Mukherjee et al., 2019; Pandey et al., 2019), *C. elegans* (Godini et al., 2019), and zebrafish (Hin et al., 2020) because of the barriers in obtaining brain tissue. Although these studies had found vital genes and pathways related to AD, these studies still have limitations in explaining the pathological mechanism of human AD. In this article, GSE118553 and GSE131617 were generated from *Homo sapiens*, so the results can better reflect the pathological mechanism of human AD patients. Moreover, we compared the expression of hub genes from modules associated with AD in the TC, FC, EC, and CE among AD and control groups. The Venn plot (Figure 4) showed that the hub genes found in the four tissues basically did not overlap, indicating that the relationship between the level of these genes and AD is different in the four tissues. Two common DEGs were found in the four tissues. The SLC6 gene family consists of four subfamilies including monoamine, GABA, amino acid, and amino acid/orphan subfamilies (Hahn and Blakely, 2007). *SLC6A12* belongs to the GABA subfamily and encodes BGT1, which is the transporter of gamma-aminobutyric acid (GABA) (Lehre et al., 2011). It is reported that the upregulation of BGT1 and downregulation of GABA signaling components are common in post-mortem human middle temporal gyrus (MTG) in AD, which may induce a cognitive decline in AD (Govindpani et al., 2020). Similar to a previous study, we found that *SLC6A12* is upregulated in AD compared with the control in TC tissue (Supplementary Table 3). Additionally, a previous study demonstrated that betaine is transported by GAT2/BGT-1 and reduces the risk of cognitive impairment in mice injected with Aβ25-35 (Ibi et al., 2019). Therefore, we speculated that *SLC6A12* may be involved in the occurrence and development of AD through the BGT-1. Moreover, several members of the SLC6 gene family are associated with the transport of GABA, taurine, and norepinephrine and may be correlated to several diseases, including the occurrence of brain diseases, especially neurodegenerative diseases (Hu et al., 2020). For instance, a previous study reported that the SLC6A3 9 repeat allele is significantly related to the genetic susceptibility of AD (Fehér et al., 2014). However, there are few reports on the role of P8 in neurodegenerative diseases except for the report that p8 may play roles in the development of the CNS in *Xenopus laevis* (Igarashi et al., 2001). In addition to the CE tissue, in the remaining three tissues, the expression of *P8* in the AD group was upregulated compared with the control group (Supplementary Table 3). Therefore, there were a few genes with a similar expression pattern among AD and control groups in four tissues. However, more data and experiments are needed to verify our findings.

The main symptom of patients with AD is cognitive dysfunction. The cognitive impairment in AD may cause synaptic dysfunction, neuronal loss, and modification of neurotransmitter receptors (Nikbakht et al., 2019). Previous work reported that abnormal synaptic and dysfunction of network synchronous activity might contribute to hippocampal-dependent memory deficits in early AD models (Mayordomo-Cava et al., 2020). According to a previous study, synaptic contacts between the neocortex and hippocampus in the brain of AD patients are lost, which is an early event in the disease process that may be involved in cognitive decline (Scheff et al., 2006). Thus, the loss of synapses is the best anatomical factor associated with cognitive deficits in AD patients (Terry et al., 1991). Moreover, hearing and sensorial impairments are prodromic symptoms of neurodegeneration, and hearing loss was reported as a risk factor for cognitive impairment and hippocampal synapse loss in AD (Chang et al., 2019).

In our study, the hub genes from modules associated with AD in the TC tissue were enriched in synapse assembly, neutrophil homeostasis, and cognition, suggesting that these genes have a high correlation with AD. The hub genes from modules associated with AD in FC tissue were involved in the regulation of postsynaptic neurotransmitter receptor internalization, indicating that the hub genes may regulate AD via the neurotransmitter receptor in the FC tissue. The hub genes from modules associated with AD in the EC tissue participated in the pathway of synaptic vesicle, suggesting that these hub genes are significant for the synaptic vesicle regulation in the EC tissue. The hub genes from modules associated with AD in the CE tissue were involved in myeloid cell homeostasis, homeostasis of several cells, and positive regulation of tolerance induction, while the module genes associated with AD in CE tissue were enriched in synapse assembly, memory, and regulation of synapse structure activity. These results revealed that the hub genes might contribute to AD pathogenesis through synapses and pathways of memory in CE tissue.

To explore the function of the hub genes in AD, we obtained expression levels of the hub genes based on Braak NFT stages from GSE131617. The results showed that the gene expression level of *PLXNB1* based on Braak NFT stages of AD in TC tissue was significant. Similarly, the gene expression level of *GJA1* based on the Braak NFT stages of AD in EC tissue was significant. These results suggested that *PLXNB1* and *GJA1* are the critical drivers in AD pathogenesis, which was supported by a previous work (Kajiwara et al., 2018; Yu et al., 2018). Additionally, we also found that GRAMD3 can distinguish Braak NFT stage in AD samples from EC tissues, which can explain that it may be involved in early cognitive decline symptoms through cerebrovascular disease (Dubé et al., 2013). However, we found that the FDR values of the three genes discussed above were greater than 0.05, suggesting that they have limitations in the classification of the Braak NFT stage in AD samples.

Machine learning combined with MRI has been proven to contribute to diagnosing several neurodegenerative diseases, including dementia (Castellazzi et al., 2020). Thus, we constructed AD classifiers, based on gene expression data of significant hub genes, which were used to explore the classification function of the genes. The results suggested that AdaBoost is the worst classifier for AD in the four tissues. The AD classifiers with the highest AUC in the TC and EC tissue were AD MLP classifier (average of AUC = 0.97 ± 0.05) and AD SVM classifier (average of AUC = 0.99 ± 0.00), respectively. The average AUC of AD classifiers in the TC and EC tissues was much higher compared with the other two tissues, suggesting the important involvement of the corresponding significant hub genes in AD pathogenesis. A total of six metrics were previously introduced to evaluate the performance of prediction methods (Vihinen, 2012), which were also used in our study. Similar to AUC of AD classifiers, the F1 score, sensitivity, specificity, PPV, and NPV of AD classifiers were higher in TC and EC tissues compared with corresponding metrics of AD classifiers in FC and EC tissues. Using fewer features in the classification model will make the model simpler but also prone to underfitting. Conversely, using too many features in the classification model will make the model complicated and overfitting (Lever et al., 2016). The good performance of AD classifiers in TC and EC tissues may be due to the selection of appropriate significant hub genes. In conclusion, the significant hub genes identified in the modules associated with AD in the TC and EC tissues have good AD classification ability, worthy of further study.

Although this study discovered some genes that may be related to the occurrence and progression of AD and built an AD machine classifier on this basis, our research conclusions still have certain limitations. All results in this study were based on public data and published results, and have not been verified by biological experiments or clinical observations. We used AD-related hub genes to construct an AD machine classifier, and it turns out that most AD machine classifiers have AUC values above 0.9; however, the number of training samples and test samples we used is too small, which may lead to overfitting of the AD classifiers. In the future, we will verify our findings through more carefully designed experiments. Meanwhile, more AD datasets will also be included in the training and testing of AD machine classifiers.

## Conclusion

In conclusion, our study identified 33 and 42 hub genes from modules associated with AD in TC and EC tissues. Among them, *PLXNB1*, *GRAMD3*, and *GJA* were correlated with Braak NFT stages of AD, suggesting that these genes may be involved in AD pathogenesis and have a high potential for AD biomarker.

## Data Availability Statement

Publicly available datasets were analyzed in this study. This data can be found here: GSE118553 and GSE131617 were collected from the NCBI GEO database.

## Author Contributions

XL designed the study. XZ and HY performed the data analysis and wrote the manuscript. All authors read and approved the final manuscript.

## Conflict of Interest

The authors declare that the research was conducted in the absence of any commercial or financial relationships that could be construed as a potential conflict of interest.

## Footnotes

1 https://www.ncbi.nlm.nih.gov/gds

2 https://github.com/BioinformaticsMan/probeToGeneSymbol.git

3 https://cran.r-project.org/web/packages/pheatmap/index.html

4 https://github.com/BioinformaticsMan/anova.git

5 https://cran.r-project.org/web/packages/beeswarm/index.html

## Supplementary Material

Supplementary Figure 1 Results of WGCNA analysis in the TC tissue. **(A)** Sample clustering and characteristics heatmap. **(B,C)** β value selection by scale independence and mean connectivity. **(D,E)** Scatter plot of GS vs. MM of the significant module associated with AD in the TC tissue. The “cor” means the PCC between GS and MM, while the p means the *P*-value calculated by verboseScatterplot function. **(F,G)** The hub genes from the subnetwork of the significant module associated with AD in the TC tissue by Cytoscape MCODE. The red diamond represents hub genes, and the green ellipse represents the nodes in the network.

Supplementary Figure 2 Results of WGCNA analysis in the FC tissue. **(A)** Sample clustering and characteristics heatmap. **(B,C)** β value selection by scale independence and mean connectivity. **(D–E)** Scatter plot of GS vs. MM of the significant module associated with AD in the FC tissue. The “cor” means the PCC between GS and MM, while the p means the *P*-value calculated by verboseScatterplot function. **(F,G)** The hub genes from the subnetwork of the significant module associated with AD in the FC tissue by Cytoscape MCODE. The red diamond represents hub genes, and the green ellipse represents the nodes in the network.

Supplementary Figure 3 Results of WGCNA analysis in the EC tissue. **(A)** Sample clustering and characteristics heatmap. **(B,C)** β value selection by scale independence and mean connectivity. **(D,E)** Scatter plot of GS vs. MM of the significant module associated with AD in the EC tissue. The “cor” means the PCC between GS and MM, while the *p* means the *P*-value calculated by verboseScatterplot function. **(F,G)** The hub genes from the subnetwork of the significant module associated with AD in the EC tissue by Cytoscape MCODE. The red diamond represents hub genes, and the green ellipse represents the nodes in the network.

Supplementary Figure 4 Results of WGCNA analysis in the CE tissue. **(A)** Sample clustering and characteristics heatmap. **(B,C)** β value selection by scale independence and mean connectivity. **(D,E)** Scatter plot of GS vs. MM of the significant module associated with AD in the CE tissue. The “cor” means the PCC between GS and MM, while the p means the *P*-value calculated by verboseScatterplot function. **(F,G)** The hub genes from the subnetwork of the significant module associated with AD in the CE tissue by Cytoscape MCODE. The red diamond represents hub genes, and the green ellipse represents the nodes in the network.

Supplementary Figure 5 Levels of hub genes from key modules associated with AD in the TC tissue. **(A)** Levels of hub genes from darkgreen module associated with AD in the TC tissue. **(B)** Levels of hub genes from darkorange module associated with AD in the TC tissue.

Supplementary Figure 6 Levels of hub genes from key modules associated with AD in the FC tissue. **(A)** Levels of hub genes from blue module associated with AD in the FC tissue. **(B)** Levels of hub genes from skyblue module associated with AD of the FC tissue.

Supplementary Figure 7 Levels of hub genes from key modules associated with AD in the EC tissue. **(A)** Levels of hub genes from blue module associated with AD in EC tissue. **(B)** Levels of hub genes form darkred module associated with AD in EC tissue.

Supplementary Figure 8 Levels of hub genes from key modules associated with AD in the CE tissue. **(A)** Levels of hub genes from brown module associated with AD in CE tissue. **(B)** Levels of hub genes from darkorange module associated with AD in CE tissue.

Supplementary Figure 9 Volcano plot and heatmap of the DEGs identified in TC **(A,E)**, FC **(B,F)**, EC **(C,G)**, and CE **(D,H)** tissue. The log2 fold change against −log10(*adj.P-*value) of DEGs is shown in volcano plots. The significantly upregulated and downregulated genes are colored in red and blue, respectively. The expression of the top 50 upregulated and downregulated DEGs (ranked by | log2FoldChange|) are shown in heatmaps.

Supplementary Figure 10 ROC curves of ten AD classifiers based on significant hub gens from modules associated with AD in TC **(A)**, FC **(B)**, EC **(C)**, CE **(D)** tissue. The ROC curve of sixfold cross-validation is displayed in different colors, and the average ROC and standard deviation are marked in blue and gray respectively.

Supplementary Figure 11 Levels of hub genes of GSE131617 in the Braak NFT stage from the TC tissue. Each point represents a sample. The height of each point represents the amount of gene expression in the sample (after log2 conversion). The color of the point corresponds to the Braak NFT stages of the sample.

Supplementary Figure 12 Levels of hub genes of GSE131617 in Braak NFT stage from the FC tissue. Each point represents a sample. The height of each point represents the amount of gene expression in the sample (after log2 conversion). The color of the point corresponds to the Braak NFT stage of the sample.

Supplementary Figure 13 Levels of hub genes of GSE131617 in Braak NFT stage from the EC tissue. Each point represents a sample. The height of each point represents the amount of gene expression in the sample (after log2 conversion). The color of the point corresponds to the Braak NFT stage of the sample. The scripts used in this studies can be downloaded in GitHub (https://github.com/BioinformaticsMan/piplineForTranscriptomAnalysis.git).

Supplementary Table 1 The PC1 of modules identified by WGCNA.

Supplementary Table 2 The detailed information of edges in the co-weighted network of each module.

Supplementary Table 3 The detailed information of the DEGs obtained among AD and control groups in four tissues.

Supplementary Table 4 The detailed information of the data used in the AD classifiers.

Supplementary Table 5 The classification metrics of AD classifiers for TC, FC, EC, and CE tissue.

Supplementary Table 6 The results of ANOVA in the Braak NFT stage of TC, FC, and EC tissue.
