# Supplementary figures and images for: Unearthing of Key Genes Driving the Pathogenesis of Alzheimer’s Disease via Bioinformatics
Source: Front Genet. 2021 Apr 16;12:641100. doi: 10.3389/fgene.2021.641100 (PMC8085575; doi:10.3389/fgene.2021.641100)

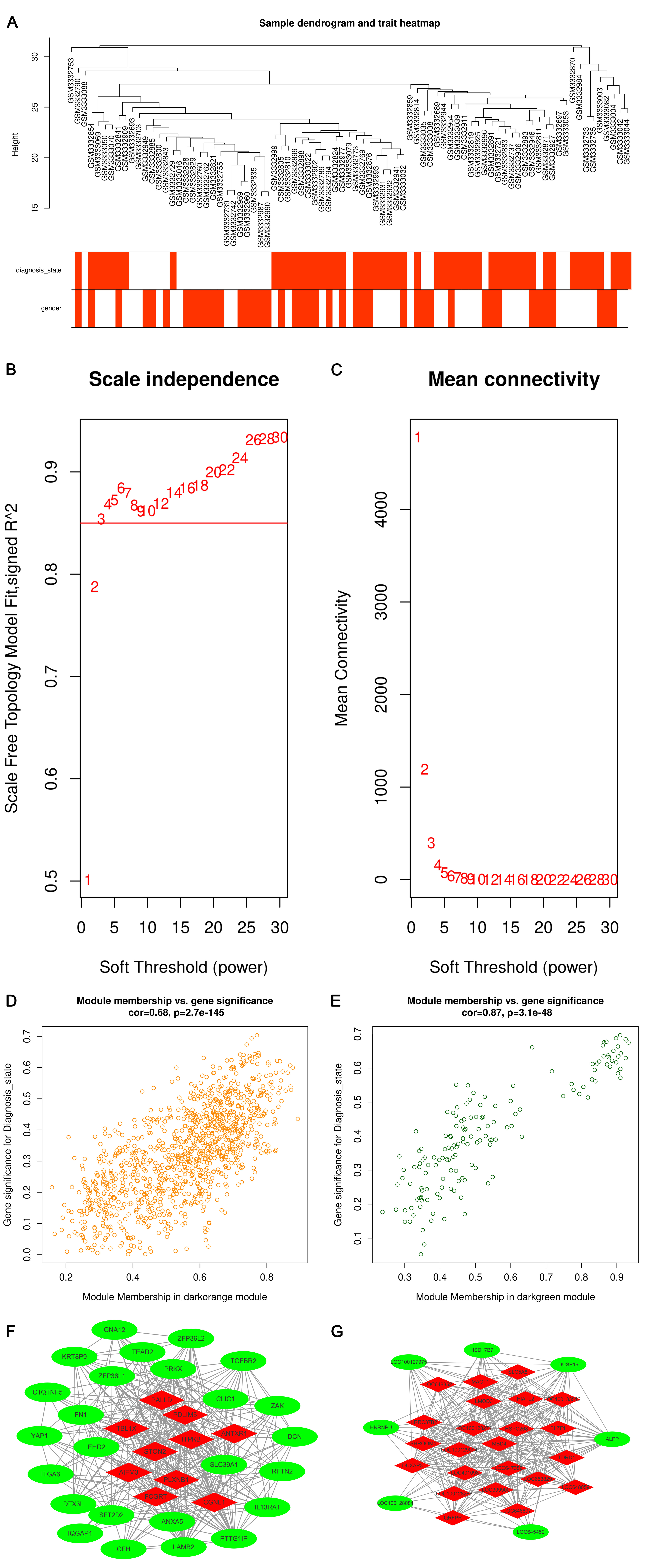

Supplement: Supplementary Figure 1 — Results of WGCNA analysis in the TC tissue. (A) Sample clustering and characteristics heatmap. (B,C) β value selection by scale independence and mean connectivity. (D,E) Scatter plot of GS vs. MM of the significant module associated with AD in the TC tissue. The “cor” means the PCC between GS and MM, while the p means the P-value calculated by verboseScatterplot function. (F,G) The hub genes from the subnetwork of the significant module associated with AD in the TC tissue by Cytoscape MCODE. The red diamond represents hub genes, and the green ellipse represents the nodes in the network. [file Image_1.TIF]

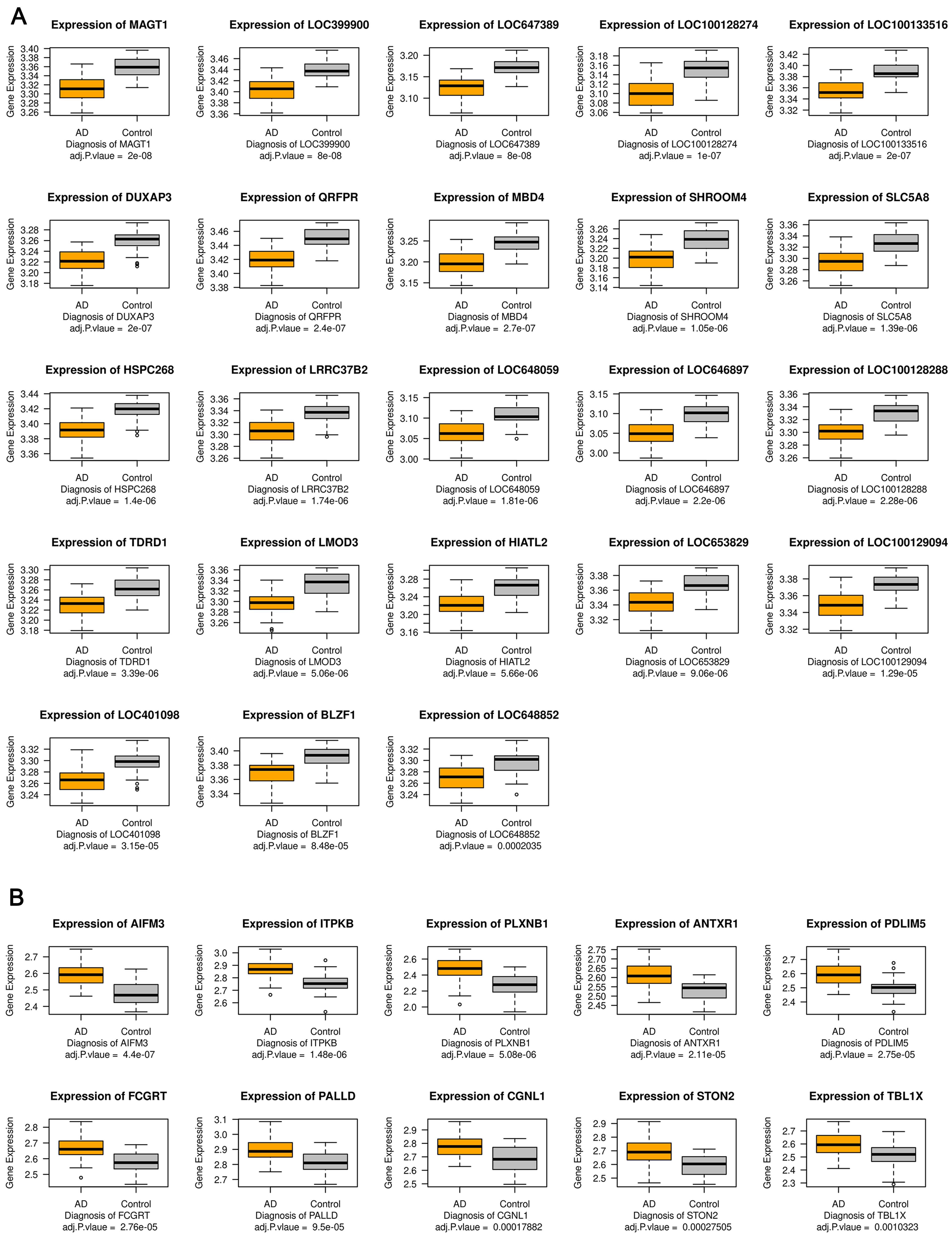

Supplement: Supplementary Figure 5 — Levels of hub genes from key modules associated with AD in the TC tissue. (A) Levels of hub genes from darkgreen module associated with AD in the TC tissue. (B) Levels of hub genes from darkorange module associated with AD in the TC tissue. [file Image_5.TIF]

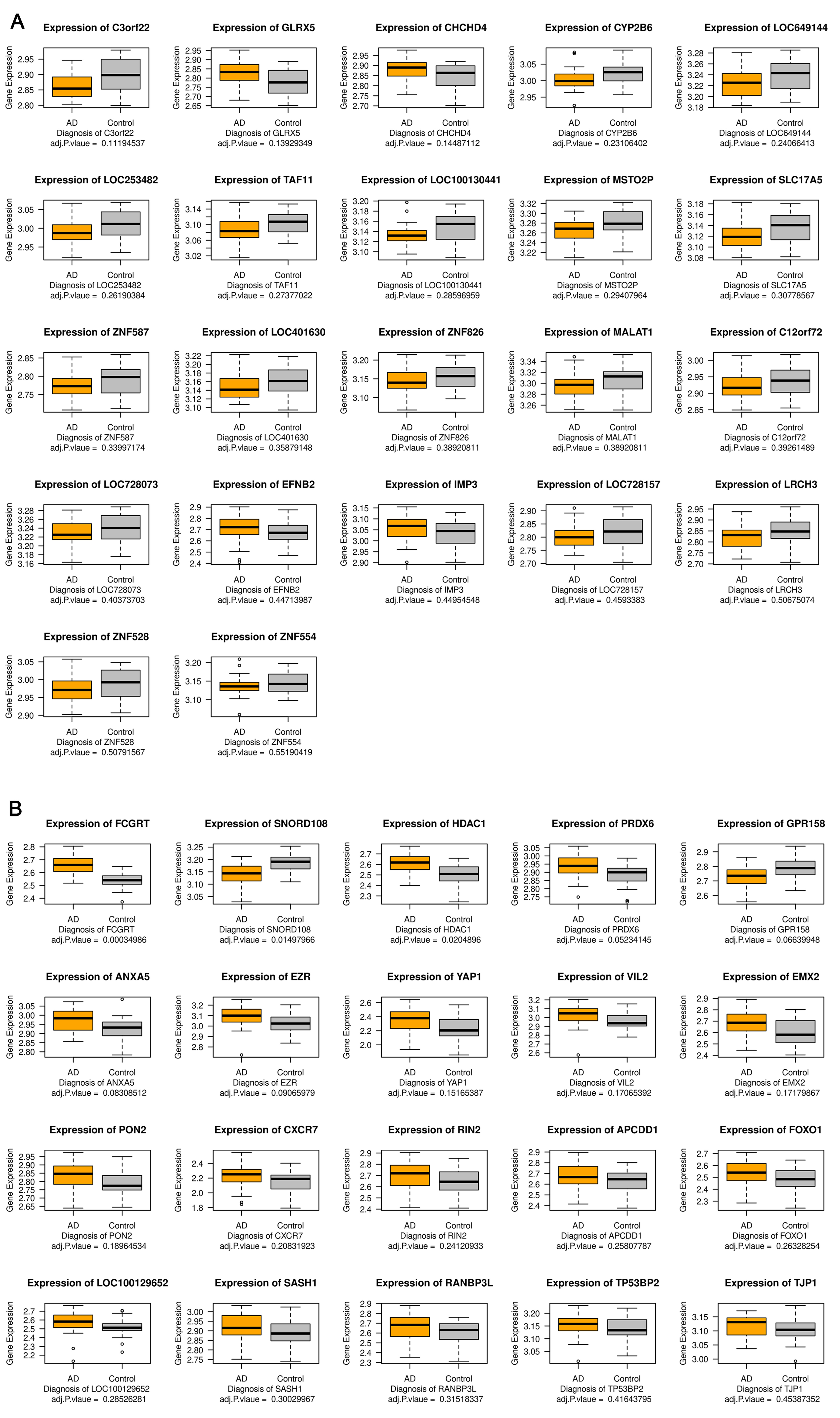

Supplement: Supplementary Figure 6 — Levels of hub genes from key modules associated with AD in the FC tissue. (A) Levels of hub genes from blue module associated with AD in the FC tissue. (B) Levels of hub genes from skyblue module associated with AD of the FC tissue. [file Image_6.TIF]

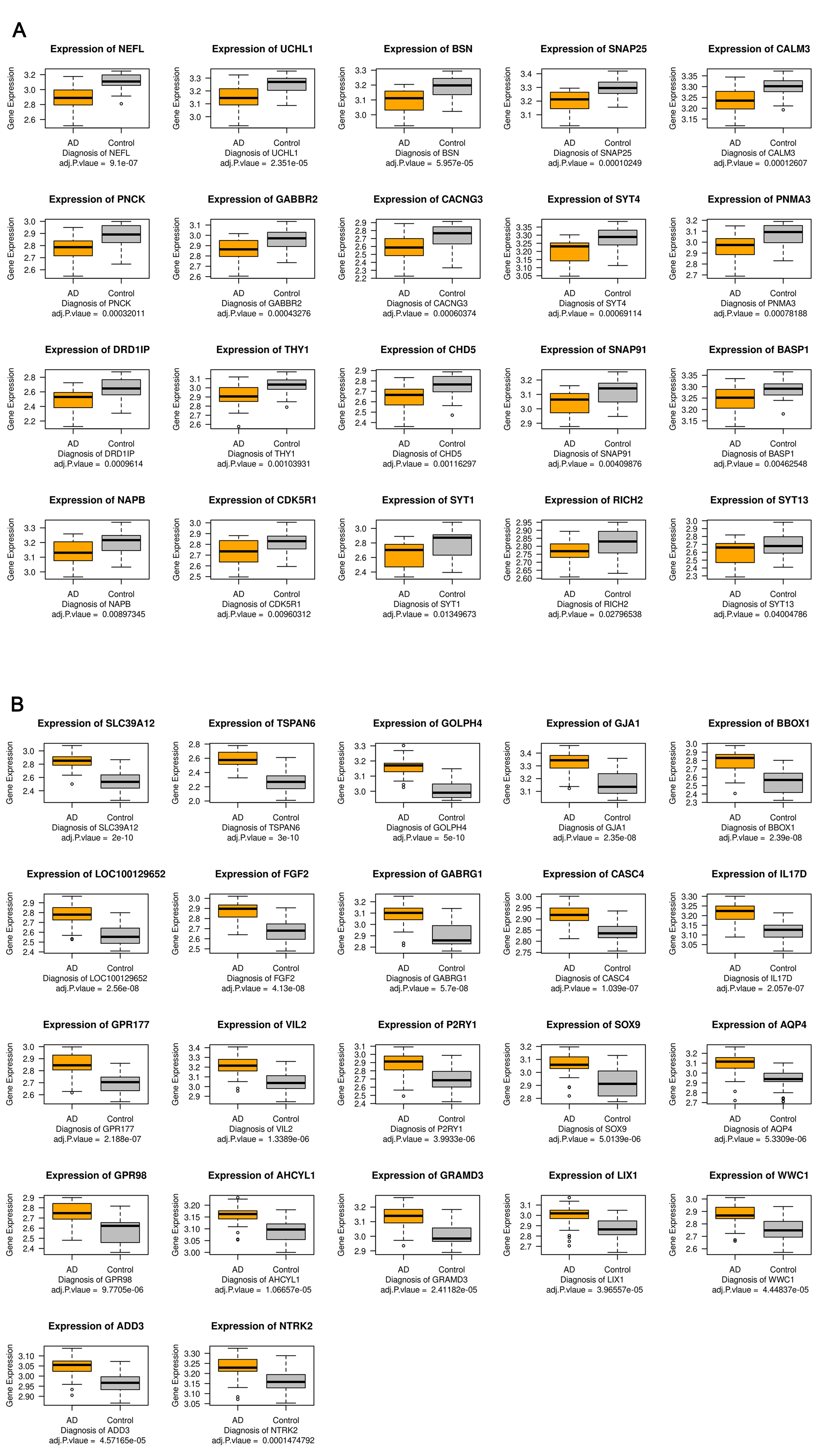

Supplement: Supplementary Figure 7 — Levels of hub genes from key modules associated with AD in the EC tissue. (A) Levels of hub genes from blue module associated with AD in EC tissue. (B) Levels of hub genes form darkred module associated with AD in EC tissue. [file Image_7.TIF]

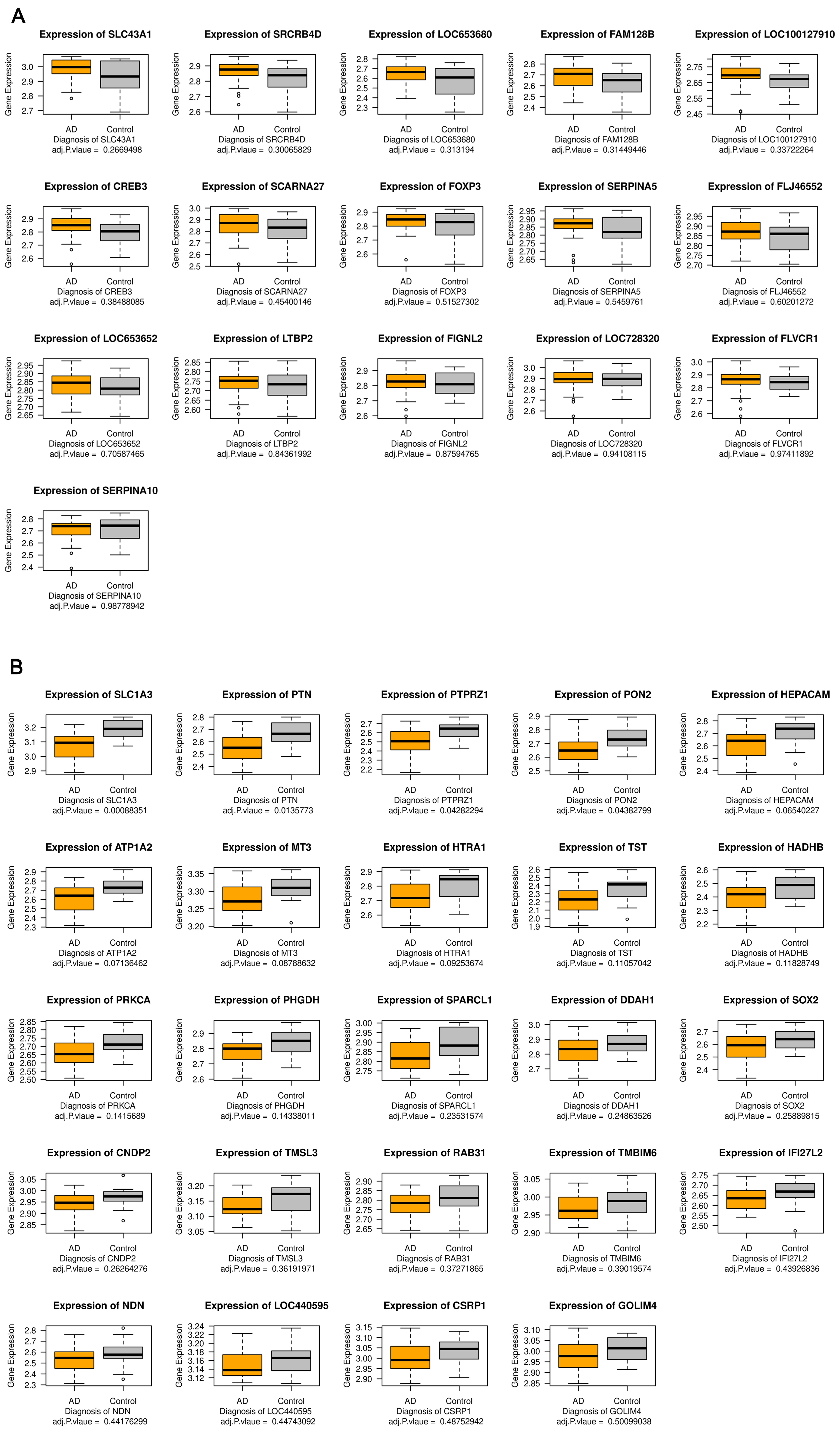

Supplement: Supplementary Figure 8 — Levels of hub genes from key modules associated with AD in the CE tissue. (A) Levels of hub genes from brown module associated with AD in CE tissue. (B) Levels of hub genes from darkorange module associated with AD in CE tissue. [file Image_8.TIF]

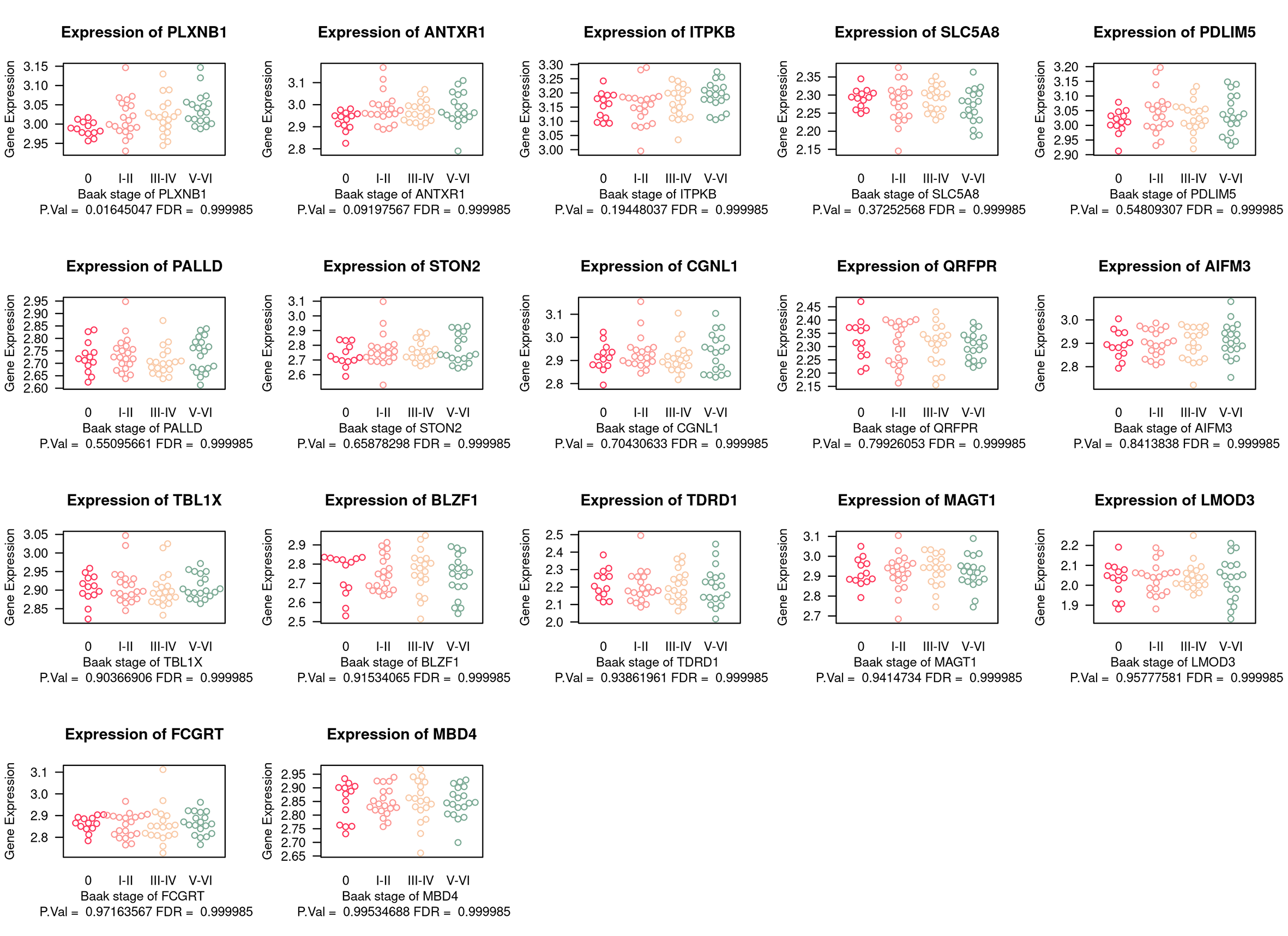

Supplement: Supplementary Figure 11 — Levels of hub genes of GSE131617 in the Braak NFT stage from the TC tissue. Each point represents a sample. The height of each point represents the amount of gene expression in the sample (after log2 conversion). The color of the point corresponds to the Braak NFT stages of the sample. [file Image_11.TIFF]

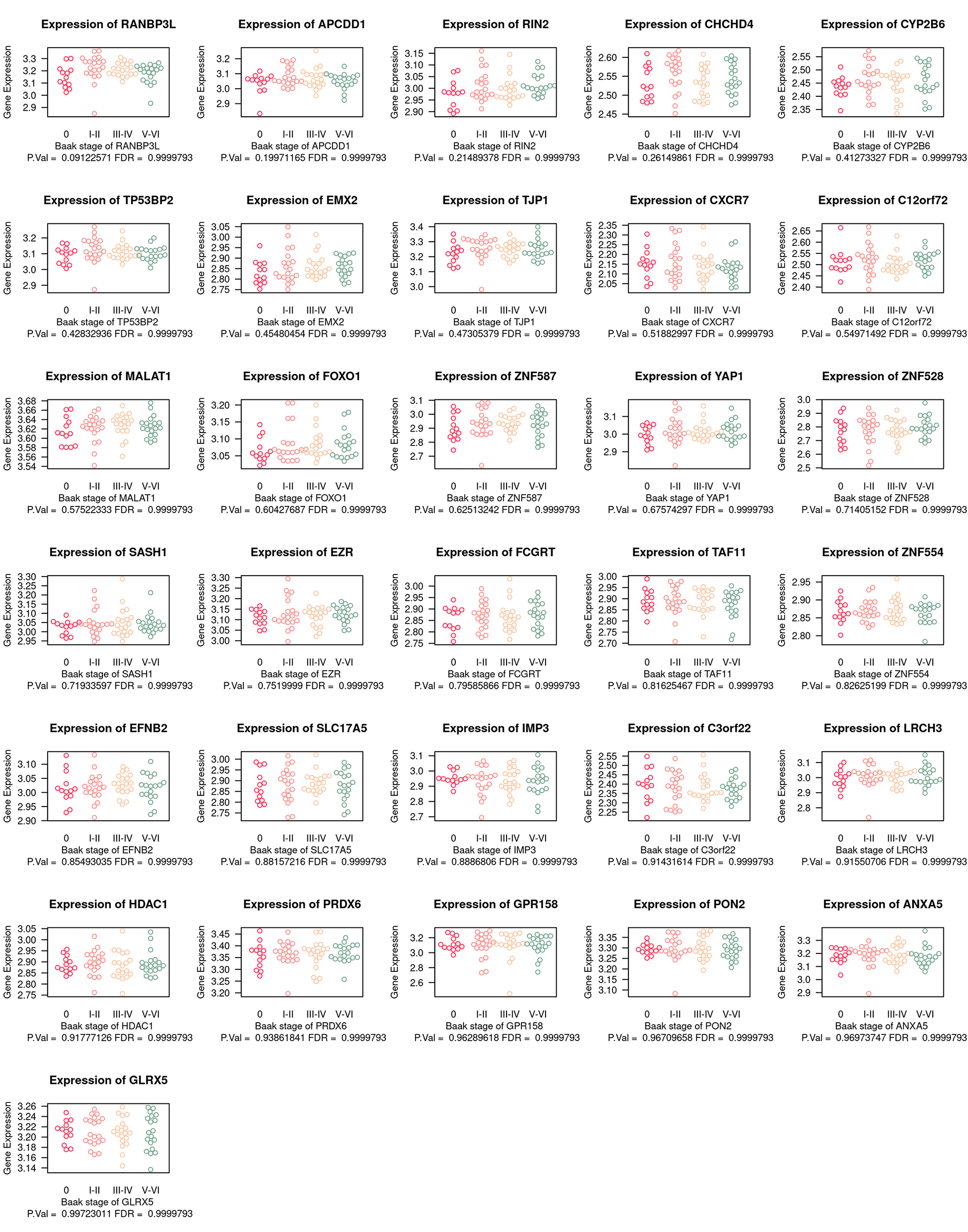

Supplement: Supplementary Figure 12 — Levels of hub genes of GSE131617 in Braak NFT stage from the FC tissue. Each point represents a sample. The height of each point represents the amount of gene expression in the sample (after log2 conversion). The color of the point corresponds to the Braak NFT stage of the sample. [file Image_12.TIFF]

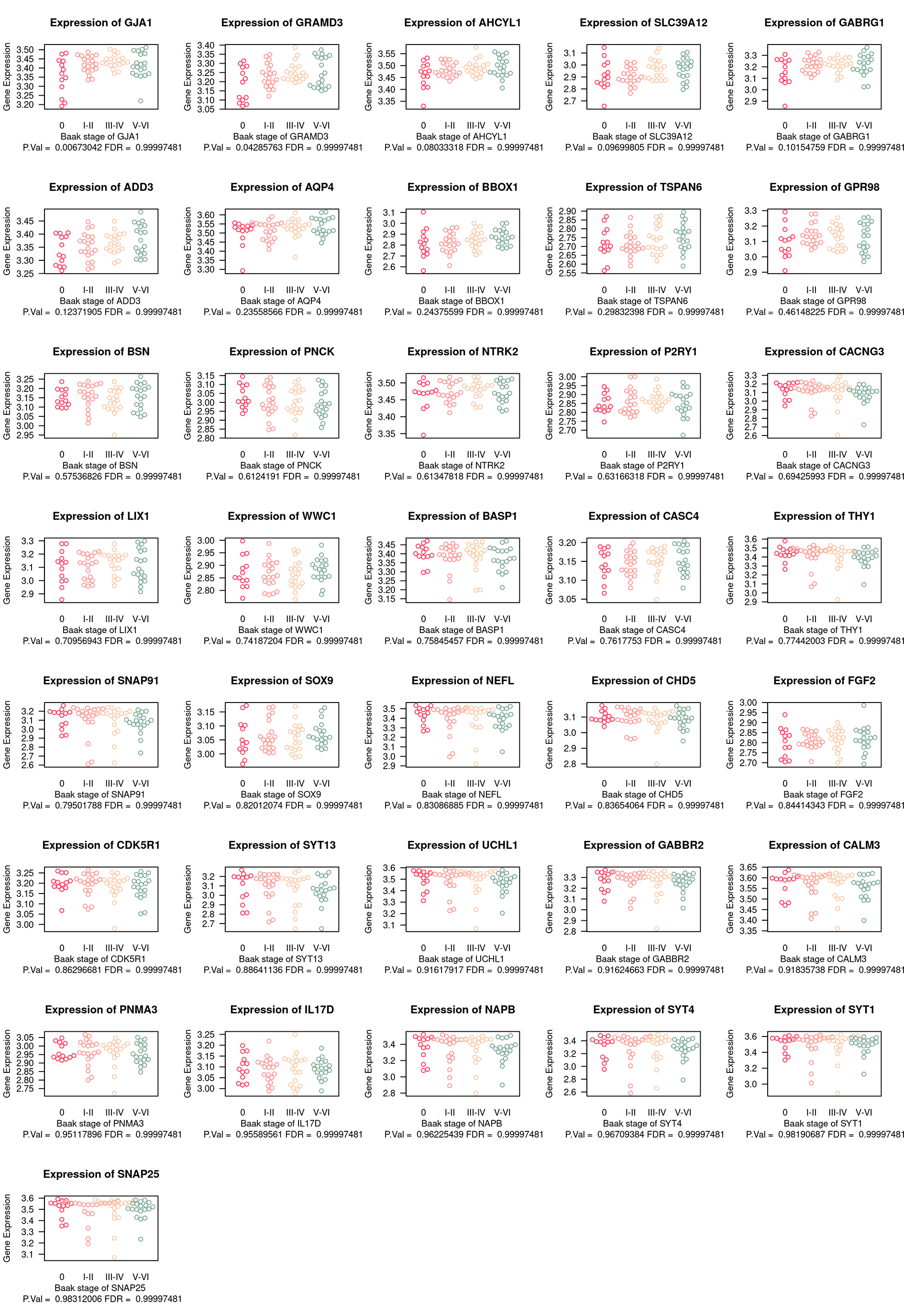

Supplement: Supplementary Figure 13 — Levels of hub genes of GSE131617 in Braak NFT stage from the EC tissue. Each point represents a sample. The height of each point represents the amount of gene expression in the sample (after log2 conversion). The color of the point corresponds to the Braak NFT stage of the sample. The scripts used in this studies can be downloaded in GitHub (https://github.com/BioinformaticsMan/piplineForTranscriptomAnalysis.git). [file Image_13.TIFF]
